# Biophysical Compatibility of a Heterotrimeric Tyrosinase-TYRP1-TYRP2 Metalloenzyme Complex

**DOI:** 10.3389/fphar.2021.602206

**Published:** 2021-04-28

**Authors:** Olga Lavinda, Prashiela Manga, Seth J. Orlow, Timothy Cardozo

**Affiliations:** ^1^ Department of Biochemistry and Molecular Pharmacology, NYU Grossman School of Medicine, New York, NY, United States; ^2^ The Ronald O. Perelman Department of Dermatology, NYU Grossman School of Medicine, New York, NY, United States

**Keywords:** tyrosinase, oculocutaneous albinism, computational molecular docking, protein-protein interface, melanosome, pigmentary disorders, molecular modeling

## Abstract

Tyrosinase (TYR) is a copper-containing monooxygenase central to the function of melanocytes. Alterations in its expression or activity contribute to variations in skin, hair and eye color, and underlie a variety of pathogenic pigmentary phenotypes, including several forms of oculocutaneous albinism (OCA). Many of these phenotypes are linked to individual missense mutations causing single nucleotide variants and polymorphisms (SNVs) in *TYR*. We previously showed that two TYR homologues, TYRP1 and TYRP2, modulate TYR activity and stabilize the TYR protein. Accordingly, to investigate whether TYR, TYRP1, and TYRP2 are biophysically compatible with various heterocomplexes, we computationally docked a high-quality 3D model of TYR to the crystal structure of TYRP1 and to a high-quality 3D model of TYRP2. Remarkably, the resulting TYR-TYRP1 heterodimer was complementary in structure and energy with the TYR-TYRP2 heterodimer, with TYRP1 and TYRP2 docking to different adjacent surfaces on TYR that apposed a third realistic protein interface between TYRP1-TYRP2. Hence, the 3D models are compatible with a heterotrimeric TYR-TYRP1-TYRP2 complex. In addition, this heterotrimeric TYR-TYRP1-TYRP2 positioned the C-terminus of each folded enzymatic domain in an ideal position to allow their C-terminal transmembrane helices to form a putative membrane embedded three-helix bundle. Finally, pathogenic *TYR* mutations causing OCA1A, which also destabilize TYR biochemically, cluster on an unoccupied protein interface at the periphery of the heterotrimeric complex, suggesting that this may be a docking site for OCA2, an anion channel. Pathogenic *OCA2* mutations result in similar phenotypes to those produced by OCA1A *TYR* mutations. While this complex may be difficult to detect *in vitro*, due to the complex environment of the vertebrate cellular membranous system, our results support the existence of a heterotrimeric complex in melanogenesis.

## Introduction

Tyrosinase (TYR) catalyzes three reactions during melanin synthesis in mammalian skin, eyes, and other organs. Melanin biosynthesis requires TYR and is enhanced by expression of the TYR homologues, tyrosinase related proteins 1 and 2 (TYRP1 and TYRP2/Dopachrome Tautomerase). Efficient maturation of the TYR protein ensures stability, enzyme activity and delivery to melanosomes where melanin is produced and deposited. Impaired TYR maturation is common to several forms of the group of genetic disorders known as oculocutaneous albinism (OCA) in humans. Mutations in the *TYR* gene itself, many of which cause protein misfolding with attendant inability to exit the endoplasmic reticulum (ER) (Berson et al., 2000; Toyofuku et al., 2001a; Chaki et al., 2010), result in OCA type 1 (OCA1A). Less severe *TYR* mutations cause partial loss of function and reduced protein folding efficiency (OCA1B) (Toyofuku et al., 2001a). TYR is retained in the ER in OCA2 (Chen et al., 2002) [*OCA2* gene mutations (Kedda et al., 1994)], OCA3 (Toyofuku et al., 2001b) [*TYRP1* mutations (Manga et al., 1997)] and OCA4 (Costin et al., 2003) [*SLC45A2* mutations (Newton et al., 2001)].

Trait-associated (non-pathogenic) *TYR* sequence variants (SNVs) that appear to exhibit maturation deficits are determinants of both skin (Shriver et al., 2003) and eye color (Sulem et al., 2007). Missense *TYR* SNVs that foster the production of autoantibodies against the protein correlate with reduced susceptibility to melanoma (Gerstenblith et al., 2010) and increased risk of the melanocyte-targeting autoimmune disorder vitiligo (Gerstenblith et al., 2007; Damico et al., 2009; Jin et al., 2010; Spritz, 2010). *TYR* SNVs are also genetically associated with susceptibility to cutaneous squamous cell carcinoma (SCC) (Asgari et al., 2016), the second most common cancer in the United States, and to its precursor, actinic keratosis (Jacobs et al., 2015). Interestingly, this association is independent of skin color (Jacobs et al., 2015; Asgari et al., 2016; Zhong et al., 2017). Over 100 *TYR* SNVs have been associated with disease phenotypes, while just a handful of variants are prevalent in the population at large. Thus, distinct phenotypes are caused by, or associated with, *TYR* amino acid variants/mutants.

TYR is a type I membrane protein, with a large intralumenal N-terminal folded domain followed by a short, flexible carboxy-terminus linker terminating in a C-terminal transmembrane helix. There are two copper binding sites required for catalytic activity, 7N-glycosylated asparagine residues and three cysteine-rich regions required for disulfide bond formation. Unlike many membrane proteins including TYRP1, TYR is retained for an unusually long period of time in the ER (Petrescu et al., 1997; Ujvari et al., 2001), which suggests that early TYR processing is highly regulated and/or complex.

The Tyrosinase family of genes is comprised of the ancestral gene *TYR* and two related genes *TYRP1* and *TYRP2*, which display a closer relationship to each other than that to *TYR* (Morrison et al., 1994). All three proteins in this family share both genetic sequence and structural similarities: all are type I membrane protein, with a large intralumenal N-terminal folded domain followed by a short, flexible linker terminating in a C-terminal transmembrane helix. In fact, TYRP1 is hypothesized to have evolved from TYR by duplication, later giving rise to TYRP2 (Sturm et al., 1995).

A number of studies suggest that TYR and TYRPs may hetero-oligomerize. Biochemically (*in vitro*), immunopurified TYR was stabilized and more active with immunopurified TYRP1 and/or TYRP2 (Hearing et al., 1992; Kobayashi et al., 1994; Winder et al., 1994), and gel filtration chromatography experiments demonstrated that TYR and TYRP1 heterodimerize in solution (Jiménez-Cervantes et al., 1998). In cells, co-expression of TYRP1 or TYRP2 (TRPs) has been shown to improve TYR stability and pigmentation (Kobayashi et al., 1998; Manga et al., 2000), and chemical-crosslinking in cells suggests the association of TYPR1 with TYR (Kobayashi and Hearing, 2007). TRPs are also detected in large molecular complexes in melanocyte lysates (Orlow et al., 1994). Conversely, Lai et al. failed to detect oligomerization with *in vitro* with recombinant forms (Lai et al., 2017), and a study showed that TYR and TYRP1 may have a low propensity to form oligomeric complexes in *in vitro* conditions designed to mimic the *in vivo* environment of the melanosome (Dolinska et al., 2020). Thus, the question of whether TYR and the TRPs form physiologically relevant functional complexes in the ER or melanosome or both remains an unanswered question in the field.

3D structure often encodes specific, yet elusive, *in vivo* constraints. For example, the chemistry and shape of a direct protein interaction surface with a binding partner that is critical for organism fitness will be conserved through evolution, even if it is a transient association in a processing sequence or dependent on a large complex of allosteric cofactors, such as bridging proteins, cytoskeletal structures, membranes, carbohydrates etc. Thus, such an interaction may be difficult to reconstitute *in vitro* or even in cells, but will be evident by direct physics based complementarity between the 3D shapes of the binding partners, including for transient complexes (Medina et al., 2008). Thus, physiologically-relevant complexes may be evident in computational biophysical studies, such as computational molecular docking, that elude experimental biophysical experiments (e.g., crystallography, surface plasmon resonance) or experimental biochemical experiments (e.g., purified recombinant binding, chromatography, immunoprecipitation). We hypothesized that transient TYR complexes may be detectable by computational molecular docking, despite the difficulties of observing them biochemically. Mapping of *TYR* missense variants on TYR 3D structure in any suitable context suggested by the presence of its associated homologues TYRP1 and TYRP2 might validate such computational biophysical models and illuminate TYR function. While the 3D structure of human TYR has not been available, an X-ray crystallographic structure of closely related human TYRP1 was resolved (Lai et al., 2017). Accordingly, we built precise, full-length 3D structural models of human TYR and its homologue TYRP2, investigated how compatible, by computational molecular docking in physics-based force-fields, the 3D structural models of TYR, TYRP1, and TYRP2 were as heterocomplexes, and mapped the known phenotypic SNVs and pathogenic mutations onto the models to see if any physically realistic heterocomplexes were compatible with mutation locations. The results strongly suggest the existence of a membrane-associated heterotrimeric TYR-TYRP1-TYRP2 complex, exhibiting an unoccupied protein surface decorated with OCA1A mutations.

## Materials and Methods

### Previously Reported Mutations in TYR

Previously reported mutations in *TYR* were collected from the OMIM database: https://www.omim.org, dbSNP: https://www.ncbi.nlm.nih.gov/snp, UniProt: https://www.uniprot.org, and GWAS databases: https://www.gwascentral.org, and https://www.genome.gov databases.

### Homology Models of TYR and TYRP2

The 2.35 Å crystallographic structure of TYRP1 (PDB: 5M8L) was used as a template for homology modeling (Lai et al., 2017). Maximally-accurate, full-length 3D homology models of TYR and TYRP2 were built as previously described (Cardozo et al., 1995; Martinez-Ortiz and Cardozo, 2018). Briefly, a sequence alignment between either the human TYR enzymatic domain amino acid sequence or the human TYRP2 enzymatic domain amino acid sequence and amino acid sequence of human TYRP1 from the template structure was generated using zero-end-gap global alignment, which is a variation of the original Needleman-Wunsch dynamic programming global alignment optimized for structural homology detection, and the *p*-value for statistical confidence/strength of this alignment was calculated (Abagyan and Batalov, 1997). The few TYR amino acid residue assignments predicted by the alignment to be at or in the vicinity of inserted or deleted residues relative to the two proteins were adjusted manually based on the 3D structural environment, as previously described (Cardozo et al., 1995), which resulted in significant conformational differences in these areas between our models and models generated previously by Lai et al. (e.g., Supplementary Figure S1). The conformations of non-identical side chains and loops containing insertions or deletions were then predicted *ab initio* using the Biased Probability Monte Carlo (BPMC) algorithm (Abagyan and Totrov, 1994). Energy-based refinement was carried out until minimal energy was reached, producing the most unclashed, stable, relaxed models possible for the purposes of protein-protein docking. As with the TYRP1 model used as a template modeling (Lai et al., 2017), glycans were not included in the models. Although some studies suggest that glycans are important for TYR stability, these studies used mutagenesis of glycosylated asparigines and did not prove that observed loss of stability was due to deglycosylation or the mutations themselves, which are historically far more deleterious to protein structure than deglycosylation (Cai et al., 2017; Dolinska and Sergeev, 2017). All modeling procedures were carried out using ICM-Pro/Homology software (Molsoft LLC, La Jolla, CA).

### Prediction of Protein Interaction Sites on the Surface of TYR

Potential protein interfaces on TYR and TYRP2 were predicted using optimal docking area (ODA) (Fernandez-Recio et al., 2005b). The same analysis was performed for the TYRP1 structure as part of the analysis of the complexes of TYR, TYRP1, and TYRP2.

### Protein-Protein Docking

Docking to the TYR homology model (assigned as a receptor) using either the TYRP1 crystal structure or the TYRP2 homology model as rigid body search models/ligands was performed as previously described (Fernández-Recio et al., 2005a). Briefly, a set of soft receptor potentials were pre-calculated using the atom coordinates of the TYR model on a 0.5 A grid from realistic solvent-corrected force-field energies, including improved implementations of de-solvation. A rotational and translational Monte-Carlo search with local minimization of the rigid all-atom model of either TYRP1 or TYRP2 within these potentials was then performed. This search finds the correct solution as the lowest energy conformation in almost 100% of test cases in which interfaces do not change on binding (Fernández-Recio et al., 2003; Fernandez-Recio et al., 2005a). The global optimization of the interface side-chains of up to 400 of the best solutions was then performed, in order to improve accuracy for cases where the interfaces change somewhat upon binding. Solutions were then ranked according to the energy terms, which include van der Waals, solvation electrostatics, hydrogen bonding and entropy. Once the heterodimers were identified and optimized, each heterodimer was then docked to the third protein in the Tyrosinase family, i.e., TYR-TYRP1 dimer was docked to TYRP2 and independently TYR-TYRP2 dimer was docked to TYRP1. This approach was taken to ensure reproducibility of the docking approach. If the heterotrimer complex obtained in the first docking procedure is valid, we expected it to be reproducible by docking using the second approach. Both docking protocols converged on the same lowest energy heterotrimer structure, within which the TYRP1 and TYRP2 conformations did not clash with each other despite being unaware of the other during docking.

### Model of Transmembrane Helix Bundle and Linkers Between Transmembrane Helices and Enzymatic Domains

The template (HIV gp41 PDB 3F4Y) for modeling a parallel, transmembrane three-helix bundle formed by the three Tyrosinase family proteins in a complex was identified using the CC+ database (Testa et al., 2009). The sequences of the C-terminal transmembrane domain of TYR (amino acids 477-497: WLLGAAMVGAVLTALLAGLVS), TYRP1 (478-501: IIAIAVVGALLLVALIFGTASYL) and TYRP2 (473-493: LLVVMGTLVALVGLFVLLAFL) were modeled on the central parallel three helix bundle from the template using the same homology modeling approach as for the enzymatic domains. This 3-helix bundle was placed equidistant from the three C-termini of the three models in the heterotrimeric complex at a distance suitable for the shortest linker to reach between the two and the linkers were energy minimized.

## Results and Discussion

### Models of TYR-TYRP Heterocomplexes: Homology Models

In order to determine if TYR and the TRPs form molecular surfaces that are complementary to each other in pairs or trimers in terms of shape, electrostatics, and other physics-based properties (hydrophobic effect in water), we built maximally accurate, energy minimized models of TYR and TYRP2 based on their homology to the TYRP1 crystal structure (PDB: 5M8L; resolution 2.35 A) (Lai et al., 2017). We recently demonstrated potential inaccuracies in models generated by the most commonly used homology modeling methods (Martinez-Ortiz and Cardozo, 2018). The state-of-the-art method requires the use of zero-end-gap global alignment with an associated *p*-value for structural similarity (Abagyan and Batalov, 1997) to identify the best possible starting target-template sequence alignment, which is then adjusted according to the structure. The sequence alignment between the TYR residues 1–510 and the corresponding intramelanosomal domain sequence from the crystallographic structure of TYRP1 (residues 25–471) was calculated to have highly significant 3D structural similarity [sequence identity 45%, *p* = 10^−40^ (Abagyan and Batalov, 1997), Figure 1]. Based on prior studies, the quality of a homology model built from a sequence alignment of this strength is at least as good as an experimentally determined structure determined with NMR spectroscopy techniques or comparable to an X-ray crystallographic structure of moderate resolution (e.g., 3.5 Å) (Cardozo et al., 1995; Abagyan and Batalov, 1997; Abagyan and Totrov, 1997; Timothy et al., 2000). Accordingly, we built these maximally accurate homology models of TYR and of TYRP2 (residues 1–519), which also shares a high structural similarity with TYRP1 (sequence identity 54%, *p* = 10^−48^).

**FIGURE 1:**
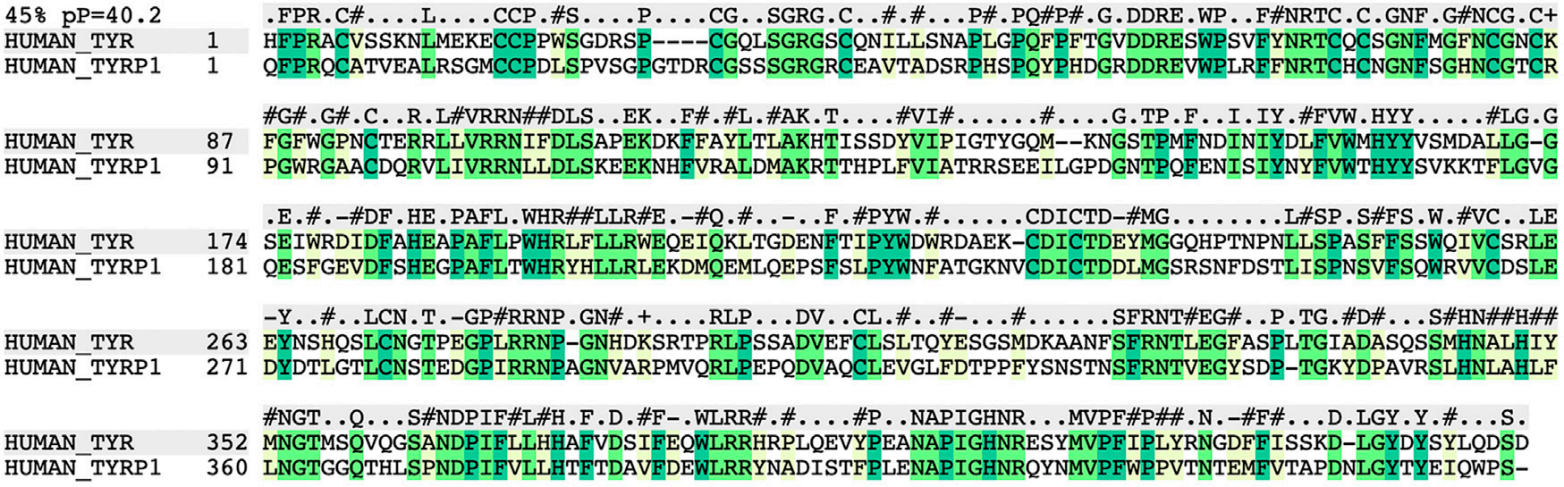
Global Needleman and Wunsch sequence alignment between TYR and TYRP1 (PDB: 5m8n) showing 45% homology with a *p*-value (*p* = −log of pvalue) of 10^−40.2^ for structural similarity. Residues highlighted in green represent conserved residues. Residues highlighted in yellow represent non-identical but homologous residues, which are expected to have little to no effect on the overall structure of the protein.

### Protein-Protein Docking

Because we were able to generate maximally accurate, low-energy 3D models of TYR, TYRP1 and TYRP2, we hypothesized that a computational molecular docking algorithm, which is customized for docking of pairs of protein domains and have proven highly successful in the CAPRI blind prediction tests (Fernández-Recio et al., 2003; Fernandez-Recio et al., 2005a), could be used to dock the TYRP1 model with the structure of TYR. Eight energetically reasonable conformations were identified in the search. The lowest energy minimum conformation was separated by at least five energy units (approx. kcal/mol) from the other seven conformations, indicative of a highly energetically preferred conformation for the complex. This process was repeated to dock TYRP2 to TYR, and a similarly obvious result for a heterotrimer conformation was obtained.

### Models of TYR-TYRP Heterocomplexes: TYR-TYRP1 Heterodimer

**FIGURE 2:**
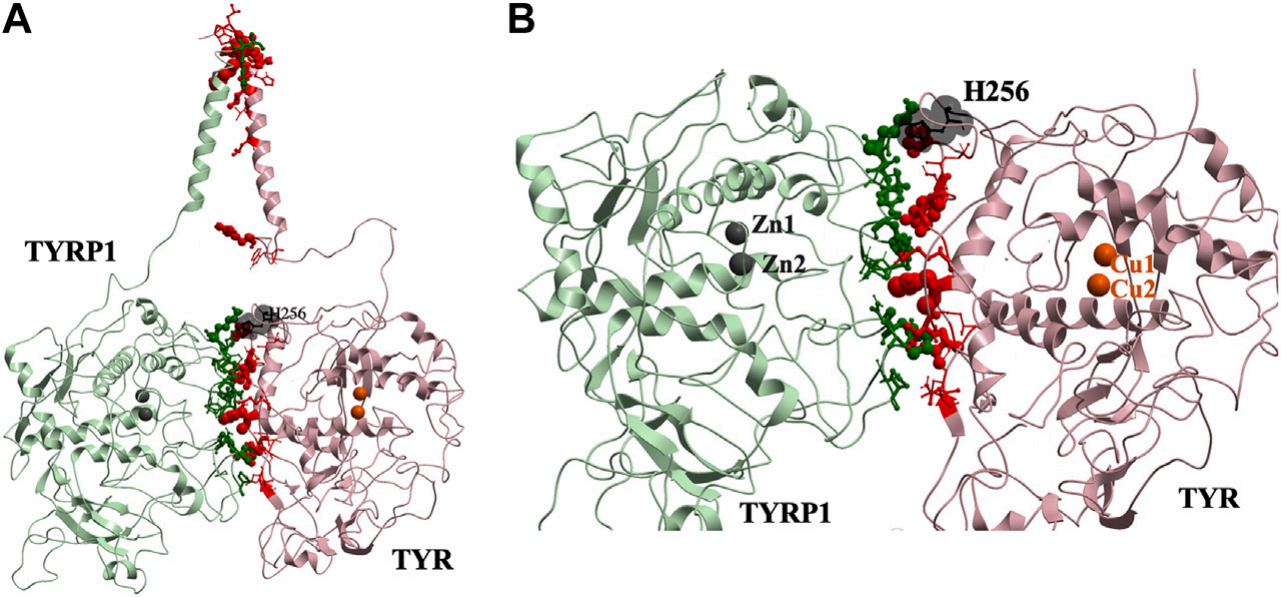
Protein-protein interaction surface between TYR and TYRP1. Residues making key contacts are displayed in ball-and-stick (radius represents contribution size). **(A)**. Full size dimer with helix bundle. **(B)**. 24 contact residues highlighted at the interface of the two proteins. H256 residue where mutation associated with OCA1A occurs, is shown in black. Two Zn atoms in the active site of TYRP1 are shown in gray. Cu atoms in the active site of TYR are shown in orange.

This docked model reveals an interaction surface between the TYR intramelanosomal domain and TYRP1 (Figure 2). 24 contact residues were identified on the surface of TYR exhibiting various strength of interactions. H256Y mutation associated with OCA1A disease also maps on the interaction surface between these two proteins (Farney et al., 2018). This SNV is predicted by PolyPhen2 to be destabilizing to the 3D structure (PolyPhen2 score 0.716): PolyPhen2 uses a machine-learned, probabilistic mathematical model to predict structurally destabilizing mutations on the basis of the evolutionary substitution pattern and the surface accessibility at that amino acid position (Adzhubei et al., 2010). The transmembrane domains of both proteins and the linkers between the transmembrane helices and the intramelanosomal domains were modeled onto this docked complex to test whether they were consistent with a transmembrane domain interaction as well.

### TYR-TYRP2 Heterodimer

**FIGURE 3:**
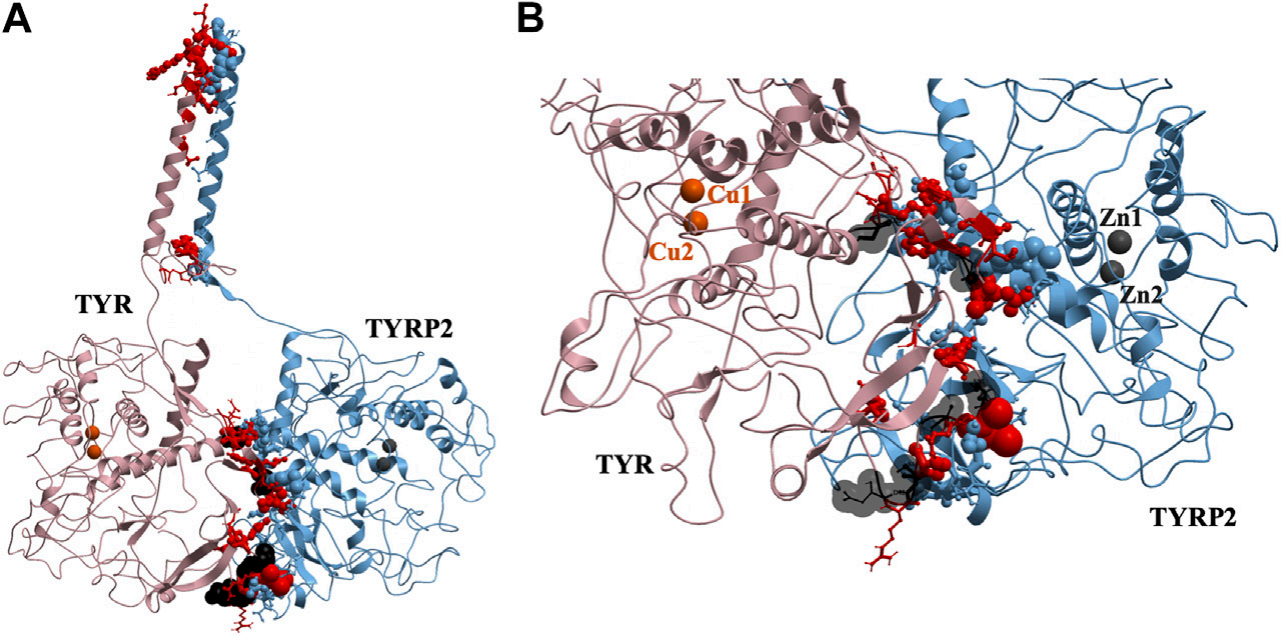
Protein-protein interaction surface between TYR and TYRP2. Residues making key contacts are displayed in ball-and-stick (radius represents contribution size). **(A)**. Full size dimer with helix bundle. **(B)**. 37 contact residues highlighted at the interface of the two proteins. Residues where mutation associated with OCA1A occurs, is shown in black. Two Zn atoms in the active site of TYRP2 are shown in gray. Cu atoms in the active site of TYR are shown in orange.

37 contact residues were identified on the surface of TYR exhibiting various strength of interactions with the surface of TYRP2 structure in a docked model of the TYR-TYRP2 heterodimer (Figure 3). Among these residues, we identified two glycosylation sites N111 and N230. Although we did not model the effects of glycosylation, the presence of the sites on the surface and specifically to the interface of protein-protein interaction surface suggests a homing or chaperone function for these glycans. In addition, six mutation sites associated with OCA1A also map at the TYR-TYRP2 interaction surface. G227 is missing in OCA1A, which could disrupt the formation of the dimer. Two polymorphic residues S44 and G47, as well as S5, G19, and D42 which have documented single nucleotide polymorphisms are all linked to OCA1A and are located at the interface with TYRP2 (Figure 3) (Simeonov et al., 2013).

### TYR-TYRP1-TYRP2 Heterotrimeric Complex

**FIGURE 4:**
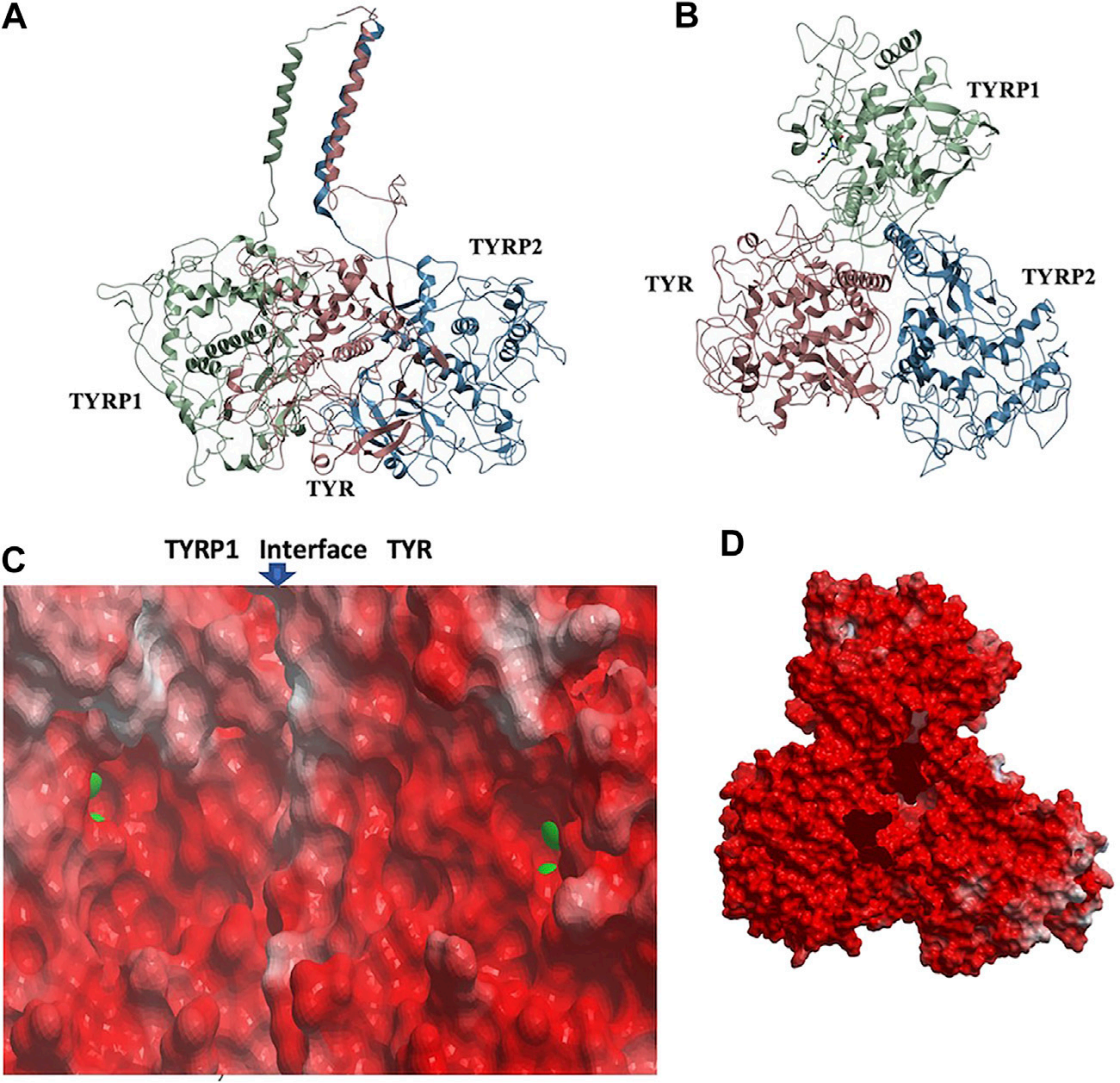
TYR-TYRP1-TYRP2 heterotrimeric complex. **(A)**. Side view, highlighting compatibility of the docked locations of the three N-terminii and their linker lengths between intramelanosomal domains and N-terminal TM-helices with formation of three-helix TM bundle. **(B)**. Top view. **(C)**. Electrostatic surfaces of the TYR-TYRP1 interface is shown, revealing continuity of active sites of TYR and TYRP1 in heterotimer (zinc ions are green spheres marking active sites) **(D).** Electrostatic surface of heterotrimer surface facing the membrane (red = electronegative/acidic, blue = electropositive/basic).

Remarkably, docking the TYRP2 structural model to the TYR-TYRP1 heterodimer and docking TYRP1 to the TYR-TYRP2 heterodimer resulted in convergence to the same conformation of a heterotrimeric TYR-TYRP1-TYRP2 complex (Figures 4A,B). In addition, the heterotrimer model was independently consistent with a parallel three-helix, coiled-coil transmembrane helix bundle, as the C-terminii of all three intramelanosomal domains were oriented in the same parallel direction by the docking. The heterotrimer placed TYR and TYRP1 active sites adjacent to each other (Figure 4C), while the TYRP2 active site was isolated facing away on the other side of the complex. The odds of this orientation occurring by random chance with an artificially docked conformation are very low as there are thousands of energetically reasonable conformations of unconstrained heterotrimer assembly of these three domains, and very few of them would be expected to place all of the C-terminal linkers pointing in the same direction and positioned so that the linker lengths could accommodate a helical bundle. All of the parallel 3-helix bundles found as templates for our model were from viruses and mediated membrane fusion, which may be significant in this case. In addition, the surface of the heterotrimer facing the membrane in the model is highly electronegative, which is consistent with the need for transmembrane anchors to oppose repulsion between this surface and the negatively charged phospholipid headgroups (Figure 4D).

The active site of TYR and its equivalent in the TYRP1 are connected by a contiguous channel, with a continuous pocket through which a substrate or a reactive metabolite may be easily shuttled between the active sites of the two enzymes (Figure 4C). Substrate/metabolite channeling has not been studied for human melanogenic proteins; however, it has been proposed to have a significant advantage for the human melanin synthesis cascade such as reduction of cellular toxicity and increase in substrate flux (Sugumaran et al., 2000; Sweetlove and Fernie, 2018). Tyrosinase family enzymes, just like many Cu and Zn containing metalloenzymes, produce highly reactive and unstable intermediates. Thus, metabolic intermediate shuttling, which prevents diffusion of the reactive metabolite into the bulk solution, can have important consequences on the melanin production cascade. Many metalloenzymes achieve substrate channeling by the formation of the multienzyme complexes. Tyrosinase produces o-benzoquinones as products of its enzymatic activity. Quinones are highly reactive electrophiles and can be toxic on cellular environment due to their reactivity (O'Brien, 1991). Although metabolite channeling is a controversial topic, available evidence suggests that complex formation would be desirable for safe processing of quinone derivatives in the cellular environment. Our trimer complex model shows that metabolite channeling is possible between TYR and TYRP1 based on their interaction surface (Table 1). The orientation of the two proteins in the complex and location of the two active sites in close proximity to each other.

**TABLE 1:** Residue contacts at the TYR-TYRP1 binding interface. Contact residues on TYR are ranked by decreasing strength of interactions, which is determined by a combination of the contact area, exposed area, the percentage of contact area compared to exposed (% buried) and closest distance to the atom on TYRP1.

**TYR residue**	**Contact area**	**Exposed area**	**% Buried**	**Closest atom**	**Mutation**	**Disease**
**H143**	95.2	144.3	66	TYRP1/pro/ca		
**F268**	69.9	125.1	56	TYRP1/pro/o		
**P265**	68.5	86.6	79	TYRP1/lys/ce		
**I145**	64.5	123.1	52	TYRP1/asn/od1		
**R504**	63.9	159.3	40	TYRP1/ala/o		
**S271**	60.0	79.4	76	TYRP1/asn/o		
**T292**	43.3	94.3	46	TYRP1/gln/o		
**Q273**	32.7	91.3	36	TYRP1/asn/nd2		
**C500**	26.2	67.3	39	TYRP1/arg/nh1		
**N168**	23.8	75.9	31	TYRP1/pro/cb		
**H256**	22.9	45.3	50	TYRP1/asp/od2	H to Y	OCA1A
**S270**	18.4	55.3	33	TYRP1/TYR/ce2		
**G291**	18.2	50.6	36	TYRP1/pro/cb		
**L262**	13.2	55.4	24	TYRP1/pro/cg		
**D169**	13.1	80.4	16	TYRP1/pro/cb		
**N171**	12.5	38.9	32	TYRP1/pro/cg		
**P293**	8.2	65.5	13	TYRP1/gln/cg		
**P257**	8.1	111.8	7	TYRP1/lys/nz		
**I170**	7.6	37.4	20	TYRP1/pro/cb		
**A266**	7.1	72.4	10	TYRP1/lys/cd		
**T258**	7.0	103.1	7	TYRP1/asp/od2		
**L140**	5.4	97.6	5	TYRP1/pro/cg		
**T144**	4.7	46.2	10	TYRP1/asn/nd2		
**I153**	3.1	62.8	5	TYRP1/pro/cb		

Interestingly, TYRP2 has a very different binding mode to TYR than TYRP1. The cys-rich subdomain (Lai et al., 2018) of TYR is involved in binding with TYRP2 in our model. The TYR-TYRP2 interaction has the larger contact area and number of residues involved (Table 2), suggestive of a higher affinity protein-protein interaction. Evidence for the evolutionary significance of the TYR-TYRP2 interaction is represented by the two glycosylation sites present in this domain.

**TABLE 2:** Residue contacts at the TYR-TYRP2 binding interface. Contact residues on TYR are ranked by decreasing strength of interactions, determined by a combination of contact area, exposed area, percentage of contact area compared to exposed (% buried) and closest distance to the atom on TYRP2.

**TYR residue**	**Contact area**	**Exposed area**	**% Buried**	**Closest atom**	**Mutation**	**Disease**
**L49**	144.0	161.4	89	TYRP2/gln/c		
**K503**	107.3	120.4	89	TYRP2/phe/cd2		
**P110**	85.3	113.1	75	TYRP2/asn/o		
**Q506**	75.9	117.2	65	TYRP2/arg/cd		
**R473**	68.3	168.9	40	TYRP2/leu/cd2		
**N111**	67.3	100.2	67	TYRP2/thr/n	Glycosylation site	
**P45**	63.2	105.9	60	TYRP2/thr/o		
**R52**	54.4	121.9	45	TYRP2/asn/n		
**L499**	52.2	104.5	50	TYRP2/phe/ce2		
**E229**	49.7	67.9	73	TYRP2/lys/cb		
**W108**	48.9	141.4	35	TYRP2/lys/o		
**R116**	44.5	106.2	42	TYRP2/lys/cd		
**W39**	43.5	187.1	23	TYRP2/val/cg1		
**E114**	42.3	102.8	41	TYRP2/TYR/ce1		
**L492**	33.0	117.9	28	TYRP2/leu/cd2		
**T113**	30.1	98.1	31	TYRP2/TYR/ce1		
**Q48**	27.4	105.9	26	TYRP2/gln/ne2		
**L507**	26.9	162.7	17	TYRP2/arg/c		
**G227**	26.2	56.2	47	TYRP2/arg/nh2	Missing	OCA1A
**S44**	24.6	67.3	37	TYRP2/asp/od1	S to G, S to R	OCA1A
**P508**	22.2	80.7	27	TYRP2/TYR/c		
**H502**	22.1	109.6	20	TYRP2/arg/nh2		
**S50**	21.5	68.7	31	TYRP2/phe/o	S to L	OCA1A
**R43**	16.8	208.0	8	TYRP2/asp/od2		
**N230**	15.3	86.0	18	TYRP2/leu/cd2	Glycosylation site	
**G101**	14.0	47.2	30	TYRP2/asn/nd2		
**V496**	11.3	83.5	14	TYRP2/phe/ce2		
**G47**	11.2	49.7	23	TYRP2/pro/cd	G to V, G to D	OCA1A
**D228**	10.5	50.5	21	TYRP2/arg/nh2		
**W477**	10.2	115.0	9	TYRP2/trp/ch2		
**K224**	9.0	159.2	6	TYRP2/pro/cb		
**G109**	6.6	27.1	24	TYRP2/TYR/oh	G to R	OCA1A
**D42**	4.8	110.3	4	TYRP2/asp/od2	D to G	OCA1A
**I474**	4.1	51.6	8	TYRP2/trp/cz3		
**T226**	2.6	41.5	6	TYRP2/leu/o		
**C112**	0.8	20.8	4	TYRP2/TYR/oh		
**E509**	0.3	157.8	0	TYRP2/TYR/cb		

### OCA1 Pathogenic Mutations Associated With ER Retention Map Onto a Unique Surface of TYR

**FIGURE 5:**
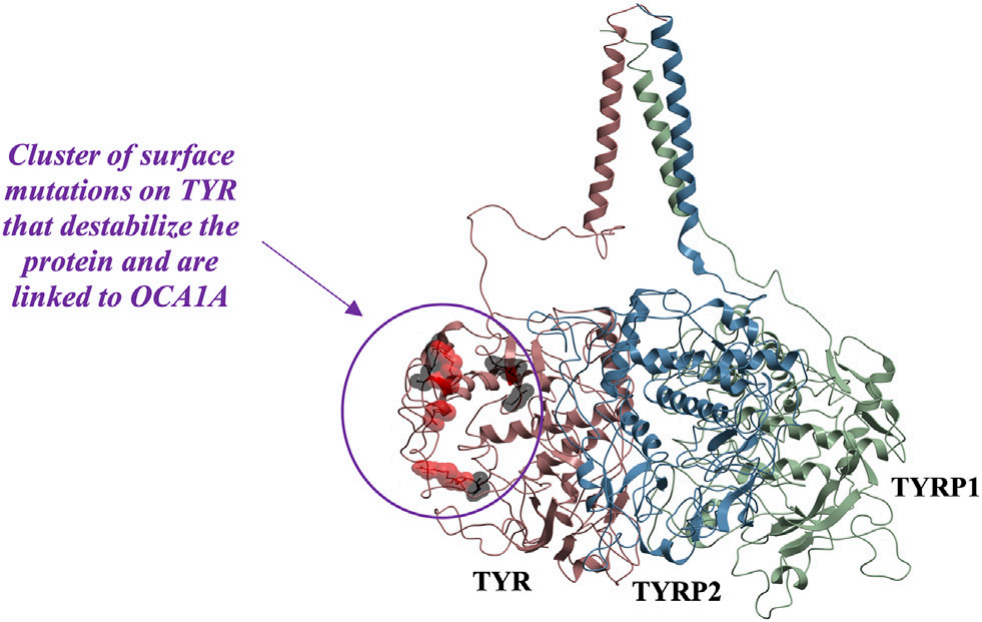
Cluster of mutations associated with ER retention map to a contiguous surface of the protein, suggesting a new interaction site with an unknown binding partner. R403, R402, P406L, R422, D448, and G446 map onto the surface of the protein in the region located distant from the active site. Substitutions shown in red (R402Q, P406L, and R422Q) result in temperature-sensitive TYR mutants.

While mapping of common mutations of TYR onto a structural model was performed previously (Lai et al., 2018) and is consistent with our model, we sought to examine mutations that have been linked to ER retention in OCA1A. Halaban et al. first demonstrated that human TYR substitutions T373K and R402Q resulted in the production of defective proteins and caused their ER retention (Halaban et al., 2000). In addition, the R402Q mutant showed temperature dependence for proper Golgi processing and transport. Toyofuku et al. expanded this list to include thermally sensitive variants P406L and R422Q (Toyofuku et al., 2001a). These mutants are functional at reduced temperatures, but lose catalytic activity at elevated temperatures, often resulting in the loss of pigmentation around warmer parts of the body. We mapped these mutations known to cause TYR retention in the ER onto the model. Mutations were color-coded to distinguish between the temperature-sensitive mutants of Tyrosinase and those that cause the ER retention but do not produce thermolabile TYR (Figure 5). The map suggests that mutations known to cause ER retention and thermal instability localize to a unique cluster on the surface of TYR.

In the past, it was postulated that these mutations result in loss of TYR catalytic activity by disrupting the copper B binding site. Yet, the R402Q and P406L variants were later shown to still be able to bind copper (Spritz et al., 1997) and as evident from the map in Figure 5, the surface localization of these mutations would make their direct effect on the active site unlikely.

**FIGURE 6:**
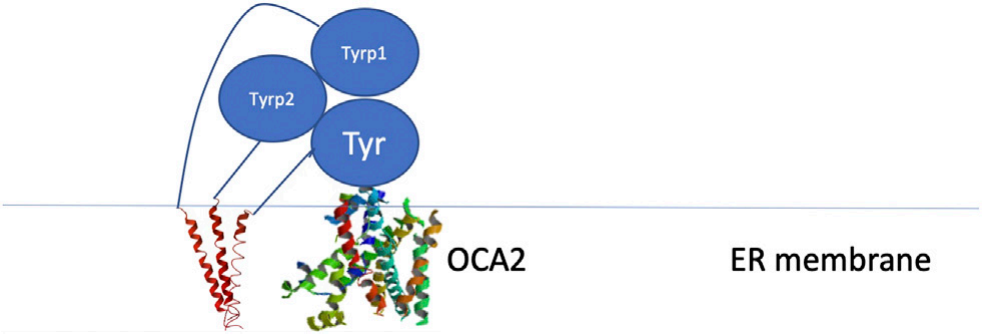
Schematic representation of the proposed site of binding between the melanogenic complex and a binding partner such as OCA2.

Because these residues localize to a surface of the protein not predicted by our model to be an interface for the TYRPs, the mechanism by which they destabilize the protein remains unclear. However, the OCA1 mutations of these residues on the surface of TYR do cause a change of net surface charge. One plausible explanation for the effect of these mutations is involvement in association with membrane phospholipids, or another currently unidentified protein partner, which would aid in the protein’s stability (Figure 6). Several binding partners for TYR have been identified, including TYRP1, TYRP2 (Orlow et al., 1994; Manga et al., 2000) as well as G Protein-Coupled Receptor 143 (De Filippo et al., 2017), while OCA4 mutations have been shown to result in reduced TYR maturation (Costin et al., 2003). Perhaps the most intriguing potential partner binding to this surface is OCA2, which is a putative anion channel. OCA2 mutations result in similar, albeit milder, pigmentary phenotypes as OCA1A and significant reduction in TYR maturation. The negative charge of the intramelanosomal heterotrimer surface suggests that the complex needs to be free to rotate and translate away from the membrane surface in order to dock this potential OCA2 surface into the membrane embedded OCA2, however such Brownian freedom would need to be constrained by the transmembrane domains. The transmembrane consistency of our heterotrimeric model thus suggests the possibility of a larger complex of this heterotrimer with OCA2, which, based on the phenotypes of mutations in the surface we have identified and the phenotype of OCA2 as preventing transit of TYR from the ER to the melanosome, may be required for transit of TYR out of the ER.

### Validation

Although monomeric TYR has been reported in purely biochemical studies (Kus et al., 2018), our model suggests that TYR and the TYRPs have evolved to form a membrane-anchored heterotrimeric TYR-TYRP1-TYRP2 complex *in vivo*. TYRP1 and TYRP2 docked unambiguously to their heterotrimeric conformations, constrained only by fundamental free energy terms, i.e., van der Waals, electrostatics, etc. If either the TYRP1-TYR or TYRP2-TYR docked conformation were artificial, clashing with the remaining TYRP docked conformation and failing to form a compatible inter-TYRP interface would be a virtual certainty. Similarly, even if an artificial docked conformation somehow survived the constraint of an independently docked counter-TYRP, the likelihood that the resulting docked conformation would also place the C-terminii of the intramelanosomal domains of all three proteins in the ideal positions to allow the linkers to form a transmembrane parallel 3-helix bundle (Figures 3–5) is very low. Finally, the consistency of all these constraints with ideal exposure of the ER retention surface in the heterotrimer (Figure 5) in the model belies an alternative molecular explanation.

## Conclusion

We built highly accurate 3D models of the structure of human Tyrosinase and TYRP2, based on the crystallographic structure of TYRP1 and comparable to experimentally determined atomic resolution structures by several informatics criteria. The models were sufficiently accurate to allow computational molecular docking to discover, using conformational search and free energy calculation alone, a model for a heterotrimeric TYR-TYRP1-TYRP2 complex that is independently consistent with voluminous independent data, including the active sites, cys-rich domain, linker and transmembrane domains of the proteins, Mendelian and pathogenic mutations and SNVs.

As noted in the Introduction, prior studies in the literature reveal a discrepancy as to whether TYR-TYRP1-TYRP2 heterocomplexes, including heterodimers of pairs of these, form. We suggest that this discrepancy derives from the inability to biochemically reconstitute the true intra-ER, transitional or intramelanosomal environment *in vitro*. The demonstration that purified intramelanosomal domains of both TYRP1 and TYR elute as monomers on size-exclusion columns and that co-elution of TYRP1 and TYR showed no heterodimer (Lai et al., 2017) by no means equates with the conclusion that there is no formation of intramelanosomal TYR-TYRP1 heterodimer or the heterotrimer proposed in this report in cells. The term “intramelanosomal” has frequently been used in these prior studies to refer to the “intramelanosomal domain” of TYR/TYRP1 (Lai et al., 2017), so basically the soluble folded domain absent the transmembrane portion and the linker. This meaning would be no different biochemically than saying “lumenal” or even “extracellular” TYR. Thus, this use of the term is somewhat misleading as these studies do not mimic the biochemical environment of the melanosome or the ER, within which physiologically relevant heterocomplex formation would take place. In addition, these studies use recombinant baculovirus expressed proteins with synthetic tags, in buffers optimized for monomer stability and crystallization, which may be very different from the *in vivo* environment. Our argument is that the *in vivo* environment is reflected more clearly in the evolution of these proteins, i.e., in their amino acid sequences. So, in our study, which relies only on this evolutionary signal, i.e., only on the biophysics of the amino acids, we see a heterotrimer. We conclude that this is evidence of the physiologically relevant *in vivo* complex, which may be hard to detect in its entirety by traditional *in vitro* biochemical means. We suspect that certain *in vitro* biochemical conditions (construct, cellular lysates, pH, concentration, temperature, etc) were ideal for heterodimer formation in the many studies leaning towards heterodimer formation and ideal for monomer formation in the conflicting studies, hence the discrepancy. The strong biophysical compatibility of evolved vertebrate TYR, TYRP1, and TYRP2 sequences, 3D structures and independent phenotype-genotype associations with a heterotrimer would be very unlikely by random chance and suggests *in vivo* relevance of the heterotrimer.

The model suggests the presence of a newly identified protein or membrane interaction surface that results in thermal instability when it is perturbed by mutation. We speculate that OCA2 may be an intriguing candidate as the binding partner for this surface and that its binding may be required for ER-melanosome transit. This model can account for the observation that TYR transit out of the ER is a “bottleneck” step of melanogenesis and requires TYRP1, TYRP2, and OCA2: the normal function of TYR that produces its baseline phenotype is highly dependent on the normal composition, architecture and sequence of ER environments, as well as ER-melanosome membrane events. The diverse pathological phenotypes associated with TYR may all have in common perturbation of this complex or of the ER environment it requires or of the membrane fusion events that transit this complex from the ER to the melanosome.

## Data Availability

The original contributions presented in the study are included in the article/Supplementary Material, further inquiries can be directed to the corresponding author.
