# Supplementary figures and images for: Biophysical Compatibility of a Heterotrimeric Tyrosinase-TYRP1-TYRP2 Metalloenzyme Complex
Source: Front Pharmacol. 2021 Apr 28;12:602206. doi: 10.3389/fphar.2021.602206 (PMC8114058; doi:10.3389/fphar.2021.602206)

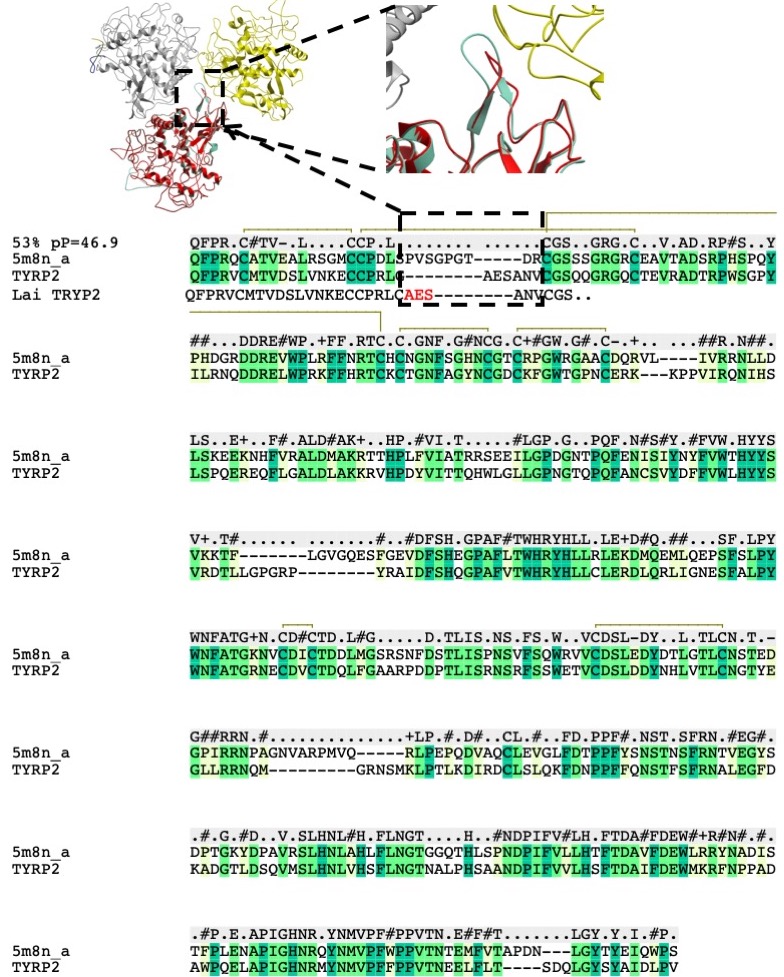

Supplement: Supplementary file 2 [file Image1.JPEG]
